# Cyclosporin A as a Potential Insecticide to Control the Asian Corn Borer *Ostrinia furnacalis* Guenée (Lepidoptera: Pyralidae)

**DOI:** 10.3390/insects13100965

**Published:** 2022-10-21

**Authors:** Chengxian Sun, Shunjia Li, Kai Wang, Xinming Yin, Yanmei Wang, Mengfang Du, Jizhen Wei, Shiheng An

**Affiliations:** 1 Henan International Laboratory for Green Pest Control, College of Plant Protection, Henan Agricultural University, Zhengzhou 450002, China; 2 College of Forestry, Henan Agricultural University, Zhengzhou 450002, China

**Keywords:** cyclosporin A, *Ostrinia furnacalis*, insecticidal activity, sublethal dose

## Abstract

**Simple Summary:**

While chemical insecticides are conventionally effective for pest control, they also cause severe problems, such as insect resistance and environmental pollution. Alternative insecticides with distinct mechanisms are needed. Cyclosporin A (CsA), a polypeptide produced by fungi, is widely used as an immunosuppressant in organ transplantation. This study used *Ostrinia furnacalis*, a major pest of maize in Asia, as a model to investigate the insecticidal activity of CsA. CsA had marked insecticide activity against *O. furnacalis* larvae. Furthermore, sublethal doses of CsA caused negative effects on larval, pupal and adult development. CsA treatments suppressed reproduction, significantly decreasing the mating rate, ovary size, and numbers and hatchability of eggs. CsA showed excellent combined toxicities when mixed with five common insecticides, respectively. Importantly, CsA acts by suppressing calcineurin, which represents a novel insecticidal target. Thus, CsA has potential as a new insecticide for sustainable pest management.

**Abstract:**

The long-term use of chemical insecticides has caused serious problems of insect resistance and environmental pollution; new insecticides are needed to solve this problem. Cyclosporin A (CsA) is a polypeptide produced by many fungi, which is used to prevent or treat immune rejection during organ transplantation. However, little is known about the utility of CsA as an insecticide. Therefore, this study evaluated the insecticidal activity of CsA using *Ostrinia furnacalis* as a model. The results demonstrated that CsA was toxic to *O. furnacalis* with LC_50_ values of 113.02 μg/g and 198.70 μg/g for newly hatched neonates and newly molted third-instar larvae, respectively. Furthermore, CsA treatment had sublethal effects on the development of *O. furnacalis*, and significantly reduced the fecundity of adults; this suggests that CsA has great potential to suppress *O. furnacalis* populations. Further analysis revealed that CsA suppressed calcineurin activity in larvae. CsA had independent or synergistic toxic effects on *O. furnacalis* when combined with β-cypermethrin, indoxacarb, emamectin benzoate, azadirachtin, and the *Bacillus thuringiensis* toxin Cry1Ac, which suggests that CsA can help prevent or manage resistance. Our study provides detailed information on the potential of CsA as an insecticide for controlling lepidopterans.

## 1. Introduction

The Asian corn borer (ACB) *Ostrinia furnacalis* Guenée (Lepidoptera: Pyralidae) is a major pest of maize throughout Asia, including in China, the Philippines, and Vietnam [1,2,3,4]. It feeds on all parts of maize (*Zea mays* L.; Poales: Poaceae), causing serious yield losses. In China, *O. furnacalis* causes average annual maize yield losses of an average of 10~20% (maximum of 30%) [5,6], making it an important corn pest.

Currently, control of *O. furnacalis* relies primarily on chemical insecticides, especially neuro-insecticides with insecticidal target sites, including allosteric sites on the nicotinic acetylcholine receptor (e.g., spinosyns), acetyl-CoA carboxylase (e.g., tetronic/tetramic acids), ecdysone agonists (diacyl hydrazines), mitochondrial electron transport chain complex II (β-ketonitrile derivatives), and an allosteric site on the ryanodine receptor (diamides) [7]. However, most of these have wide non-selective toxicity that severely threatens human health and the environment and benefits secondary pest outbreaks. For this reason, the search, design, implementation, and evaluation of new control methods play a strategic role in the effective management of this pest [8]. Widespread use of broad-spectrum, highly toxic chemical insecticides with low specificity may lead to phytosanitary risks, potentially resulting in the development of populations of resistant insects [8]. To avoid these adverse consequences, *Bacillus thuringiensis* (Bt) toxins and transgenic Bt crops that act on insect midgut receptors (including cadherin and ABC transport proteins) have been used to control *O. furnacalis* [9,10,11]. Unfortunately, more than 300 cases of insect resistance to Bt and chemical insecticides have been reported across 20 different pest insect species, including *O. furnacalis* [6,12]. Hence, insecticide resistance requires the discovery of new insecticides with novel modes of action, environmentally friendly bio-rational insecticides, and insecticidal targets [13]. 

Cyclosporin A (CsA) is a natural product mass-produced by deep fermentation of different species of fungi, including *Aspergillus terreus* Thom (Eurotiales: Aspergillaceae), *Cladosporium cucumerinum* Ellis & Arthur (Cladosporiales: Cladosporiaceae), *Penicillium fellutanum* Biourge (Eurotiales: Aspergillaceae), *Cylindrocarpon lucidum* C. Booth (Hypocreales: Nectriaceae), and many *Trichoderma* (Hypocreaceae) species [14]. CsA is used clinically to prevent or treat immune rejection during organ transplantation and various autoimmune diseases [15]. CsA is safe in humans, including pregnant women [16]. It acts by suppressing calcineurin (CaN) activity and pathways [17,18,19]. Interestingly, CsA also exhibits insecticidal activity. For example, CsA is toxic to *Culex pipiens* Linnaeus (Diptera: Culicidae) larvae [20] but not *Galleria mellonella* Linnaeus (Lepidoptera: Pyralidae) [21]. This molecule can be used as an ABC transporter inhibitor to reverse the resistance and increase ivermectin toxicity in the cattle tick, *Rhipicephalus* (*Boophilus*) *microplus* (Parasitiformes: Ixodidae) [22,23]. Although CsA may have insecticidal activity [20,24], its utility as an insecticide against agricultural pests on corn, especially *O. furnacalis*, has not been studied.

This study firstly evaluates the potential of CsA as a new insecticide for the control of *O. furnacalis* larvae in laboratory conditions. The lethal and sublethal effects of CsA on *O. furnacalis* were investigated. Considering its functions in humans, the role of CsA in controlling *O. furnacalis* was explored by measuring the CaN activity. Furthermore, the toxicity of CsA combined with β-cypermethrin, indoxacarb, emamectin benzoate, azadirachtin, and Bt toxin Cry1Ac against *O. furnacalis* was investigated. We hypothesized that CsA has good insecticidal activity against *O. furnacalis.* The findings enhance the understanding of CsA toxicity in insects and provide information that can aid in the development of new insecticides and the identification of insecticidal targets for *O. furnacalis* control.

## 2. Material and Methods

### 2.1. Insects

*O. furnacalis* was collected in Jiyuan, Henan Province, China (35.08°N, 112.57°E; elevation: 432 m), and reared for >10 generations in our laboratory at 28 ± 1 °C under a 14L/10D photoperiod with 60 ± 10% relative humidity. Larvae were fed an artificial diet [25] in a cuboid box (750 mL) until pupation. Female and male pupae were distinguished by the genital organs on the end of abdomen and placed in separate boxes. After emergence, adults (female: male ratio was 1:1.2) were transferred to cages (20 × 20 × 10 cm^3^) for mating and reproduction, and 5% sucrose solution was provided for adults to ensure adequate nutrition.

### 2.2. CsA and Insecticide Obtention

CsA (98.5% active ingredient), Cry1Ac protoxin, and azadirachtin (1% active ingredient) were purchased from Solarbio (Beijing, China), Beijing Genralpest Biotech Research (Beijing, China), and Green Gold Biotechnology (Chengdu, China), respectively. Emamectin benzoate (91% active ingredient), indoxacarb (99% active ingredient), and β-cypermethrin (95% active ingredient) were kindly provided by Bin Zhu (Department of Entomology, China Agricultural University, Beijing, China).

### 2.3. Experimental Design

To explore the insecticidal activity of CsA against *O. furnacalis*, experimental and control groups were employed for investigation, of which individuals treated with CsA comprised the experimental group, and individuals treated without CsA formed the control group. Concentrations of CsA were the design factors; mortality or effects caused by CsA were the response variables. Larvae of same size were selected for each experiment; a total of 72 larvae for three replicates (each replicate contained 24 larvae) were used for bioassay of insecticidal activity or combined toxicity of CsA with another insecticide. Baseline of the lethal toxicity was analyzed by Probit model according to the methods described by Wu et al. [26]. Combined toxicity was analyzed following the methods described by Wei et al. [27]. A total of 120 larvae for three replicates (each replicate contained 40 larvae) were selected for sublethal bioassay of CsA. Sublethal concentrations of CsA were selected based on the baseline of lethal toxicity. The larvae were fed CsA until pupation; adults were not exposed to CsA. The surviving individuals (larvae, pupae, or adults) served as sampling units for each experiment, and growth, development, and reproduction were recorded [28].

### 2.4. Insecticidal Activity of CsA against O. furnacalis Larvae

Newly hatched neonates and newly molted 3rd-instar larvae of *O. furnacalis* were selected for bioassays to test the insecticidal activity of CsA by feeding them a diet containing CsA (final concentrations of 0, 12, 24, 48, 96, 192, and 384 μg/g for neonates and 24, 48, 96, 192, 384, and 768 μg/g for 3rd-instar larvae). Equal volumes of DMSO containing different doses of CsA were fully mixed with the diet (cooled down to 42 °C). Groups of 12 neonates or 3rd-instar larvae were reared in a cup (25 mL) or Petri dish (Φ 5 cm). The artificial diet was replaced daily (72 larvae per treatment). Seven days after treatment, the LC_50_ and LC_95_ values of CsA for neonates and 3rd-instar larvae were calculated (larvae still at the 1st or 3rd instar were considered dead).

### 2.5. Effect of Sublethal CsA on Larvae, Pupae, and Adults

For the sublethal bioassay of CsA against *O. furnacalis,* newly molted 3rd-instar larvae were fed diets containing 0, 24, and 48 μg/g of CsA (LC_9_ and LC_20_) until they developed into prepupae. A Petri dish (Φ 9.5 cm) was used for rearing larvae (20/dish). Three biological replicates of 40 larvae each were performed. Seven days after CsA treatment, all live larvae were weighed on an electronic balance (BSA124S-CW; Sartorius, Göttingen, Germany). The larvae that developed into prepupae were observed every 12 h from the 3rd instar to pupal stage. After pupation, pupae from the different groups were weighed and counted to calculate the pupation rate (pupae/treated larvae). Females and males were distinguished by the abdominal genital organs to calculate sex ratios and then transferred to a box (750 mL) for emergence. The emergence dates were recorded to calculate pupal periods. When adults emerged, they were transferred to a box (750 mL) and fed 5% sucrose solution without DMSO or CsA. The total numbers of adults were recorded to calculate emergence rates (adults/pupae). Larvae, pupae, and adults were photographed with a Canon R camera (Canon, Tokyo, Japan).

### 2.6. Sublethal Bioassay on Adult Fecundity

To observe ovary development, 1-day-old (in the 1st photophase) female adults (*n* = 48 for each group) in the 0, 24, and 48 μg/g CsA-treated groups (at the larval stage) were dissected in 0.7% NaCl solution to obtain ovaries. The lengths of the ovaries and numbers of mature eggs were determined under an anatomical microscope (M205 A; Leica, Wetzlar, Germany). One-day-old female (*n* = 50 for each concentration of CsA) and male (*n* = 60) adults exposed to the different treatments (five biological replicates) were selected for a mating study conducted in 750 mL boxes. The mating rate was calculated as the number of mated females divided by the total number of females in each biological replicate (whether females mated was determined by the presence or absence of spermatheca) [29]. One-day-old virgin adult females (*n* = 60 for each concentration of CsA) and males (*n* = 72) in each treatment were allowed to mate for 1 day. Each pair was placed in a plastic box (450 mL) to investigate oviposition. When the female laid eggs, the old box was replaced with a new one, and the number of eggs per day was recorded until the female died. The neonates that hatched from eggs produced by females (*n* = 10 for each concentration of CsA) were counted to calculate the hatching rate.

### 2.7. CaN Activity Measurement

Newly molted 3rd-instar larvae were fed diets containing CsA (0, 24, and 48 µg/g). The midguts were dissected after CsA treatment for 1, 3, 5, or 7 days and then stored at –80 °C for later study. Three biological replicates were performed; each replicate contained at least 10 midgut tissues. For protein extraction, the midgut tissues were fully ground in centrifuge tubes containing 0.8% NaCl solution on ice to obtain homogenates. After centrifugation at 1500× *g* for 10 min at 4 ℃, the protein concentrations and CaN activity were determined from the supernatants using BCA Protein (Beyotime Biotechnology, Shanghai, China) and Calcineurin Activity (Abcam, Cambridge, UK) assay kits according to the manufacturers’ instructions, respectively [30,31].

### 2.8. Toxicity of CsA Combined with Other Toxins

Third-instar larvae (*n* = 72 for three replicates) were individually exposed to toxins, including CsA (final concentration in the diet of 24, 48, or 96 μg/g), β-cypermethrin (5 μg/g), indoxacarb (10 μg/g), emamectin benzoate (10 ng/g), azadirachtin (5 μg/g), and Cry1Ac (10 μg/g). Three biological replicates were used. In addition, 3rd-instar larvae were treated with different concentrations of CsA mixed with the above five pesticides. The diets used for this study were prepared according to the above method. Na_2_CO_3_ solution (50 mM, pH 10.0; dissolves Cry1Ac) and DMSO (dissolves CsA and the other four toxins) were used as control buffers. Groups of 24 larvae were reared in a Petri dish (Φ 9.5 cm) containing the diet (replaced daily). Mortality was recorded after 2 (for the combination of CsA and β-cypermethrin), 3 (for CsA and indoxacarb and CsA and emamectin benzoate), or 5 (for CsA and azadirachtin and CsA and Cry1Ac) days. The expected mortality for each combination of the two toxins was calculated following a reported method [27,32,33] as follows:

Expected mortality = (1 − S_A_ × S_B_) × 100%

where S_A_/S_B_ is the observed survival rate of larvae treated with toxin A/B. Expected mortality > observed mortality (*p* < 0.05, analyzed by independent samples *t*-test) indicates that the combined toxicity is synergistic; expected mortality < observed mortality (*p* < 0.05) indicates that the combined toxicity is antergic; expected mortality = observed mortality (*p* > 0.05) indicates that the combined toxicity is independent.

### 2.9. Statistical Analysis

LC_50_ and LC_95_ values of CsA against *O. furnacalis* neonates and 3rd-instar larvae were calculated using Probit analysis conducted with SPSS Statistics 20 software (IBM Corp., Armonk, NY, USA); heterogeneity was used in the calculation of confidence limits when the significance level was less than 0.15. The significance of multiple comparisons was analyzed at *p* < 0.05 using ANOVA followed by Tukey’s (equal variances assumed) or Dunnett’s T3 (equal variances not assumed) HSD test on SPSS Statistics 20. The dependent variables are the data on the effect caused by CsA; the fixed factors are CsA concentrations. Independent samples t-test on SPSS Statistics 20 was used to analyze significant differences of paired comparisons (expected and observed mortality of combinations of CsA with other insecticides). Expected and observed groups were the grouping variables; expected and observed mortalities were the examined variables. All shown data were means ± standard errors (SE) of at least three biological replicates.

## 3. Results

### 3.1. Insecticide Activity of CsA against O. furnacalis Larvae

The insecticide activities of CsA against neonates and third-instar larvae were analyzed according to mortality at 7 days after treatment (Table 1). The LC_50_ and LC_95_ values of CsA against the neonates were 113.02 (60.38–289.71, 95% confidence limits) μg/g and 1107.64 (381.54–38,337.57) μg/g, respectively. The LC_50_ and LC_95_ values of CsA against third-instar larvae were 198.70 (134.36–317.63) μg/g and 2457.28 (1096.90–12,480.19) μg/g, respectively.

### 3.2. Sublethal Effects of CsA on O. furnacalis Larvae

**Figure 1:**
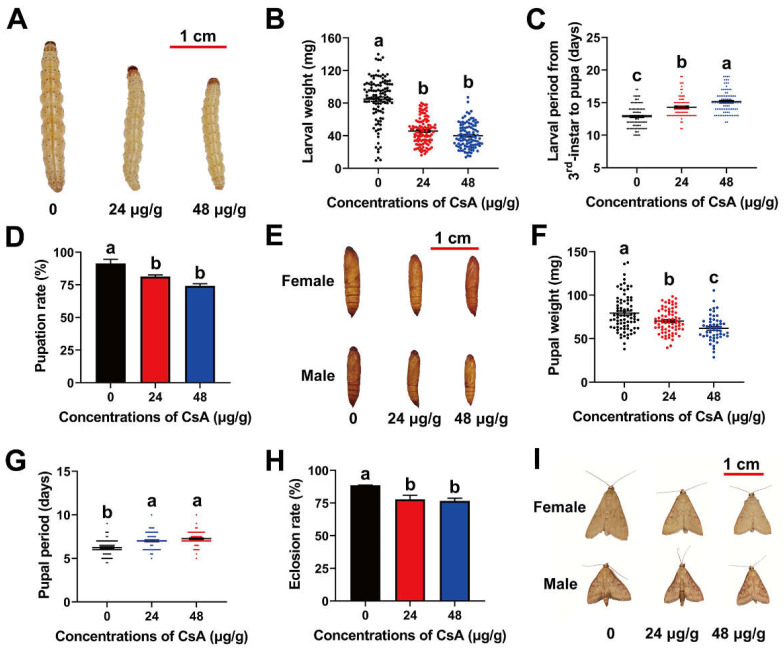
Sublethal effects of CsA on *O. furnacalis* larvae, pupae, and adults. (**A**–**C**) The body size (**A**), weight (**B**), and larval period (**C**) of the larvae after treatment with 0, 24, and 48 μg/g of CsA for 8 days. (**D**) Pupation rate under the different treatments of CsA. (**E**–**G**) Effects of CsA on the body size (**E**), weight (**F**), and period (**G**) of the pupae. (**H**) Emergence rate under the different treatments of CsA. (**I**) Phenotype of adults under each treatment. Data are means ± SE of more than three biological replicates. The significant differences were analyzed using ANOVA followed by Tukey’s (equal variances assumed) or Dunnett’s T3 (equal variances not assumed) HSD test on SPSS Statistics 20. Different lowercase letters indicate significant differences at the level of *p* < 0.05.

To investigate the effect of sublethal CsA on *O. furnacalis* larvae, third-instar larvae were fed sublethal CsA (final concentrations in diet of 0, 24, and 48 μg/g). The results demonstrated that larval development was considerably prevented compared with the control. This suppression manifested in the body size (Figure 1A), weight (Figure 1B), and development period (Figure 1C). The average weights of larvae fed 24 and 48 μg/g of CsA for 7 days were 45.72 and 39.98 mg, respectively, which are significantly lower than those of the control (84.91 mg) (F = 154.40; df = 324; *p* = 0.0001; Figure 1B). The CsA treatment significantly prolonged the mean interval from third-instar larvae to pupae from 12.86 days (control) to 14.30 days (24 μg/g) and 15.13 days (48 μg/g) (F = 45.91; df = 281; *p* = 0.0001; Figure 1C).

### 3.3. Post-Exposure Effects of CsA on O. furnacalis Pupae and Adults

The ability of larvae treated with CsA to pupate was lower than that of the control, decreasing significantly from 91.39 % (control) to 81.39% (24 μg/g CsA) and 74.17% (48 μg/g CsA) (F = 15.72; df = 8; *p* = 0.0041; Figure 1D). The CsA treatment also reduced pupal size compared with the control (Figure 1E). Correspondingly, the CsA treatment decreased the average weight of pupae from 79.35 mg (control) to 70.17 mg (24 μg/g CsA) and 59.56 mg (48 μg/g CsA) (F = 16.82, df =201, *p* = 0.0001; Figure 1F), while significantly prolonging the CsA treatment from 6.24 (control) to 7.02 (24 μg/g CsA) and 7.26 (48 μg/g CsA) days (F = 36.53; df = 256; *p* =0.0001; Figure 1G). As expected, the CsA treatment increased the rate of emergence failure from 11.5% (control) to 22.23% (24 μg/g CsA) and 23.40% (48 μg/g CsA) (Figure 1H). Similar to those of the larvae and pupae, the body size of adults decreased with the increasing CsA doses (Figure 1I).

### 3.4. Post-Exposure Effects of CsA on O. furnacalis Reproduction and Eggs

**Figure 2:**
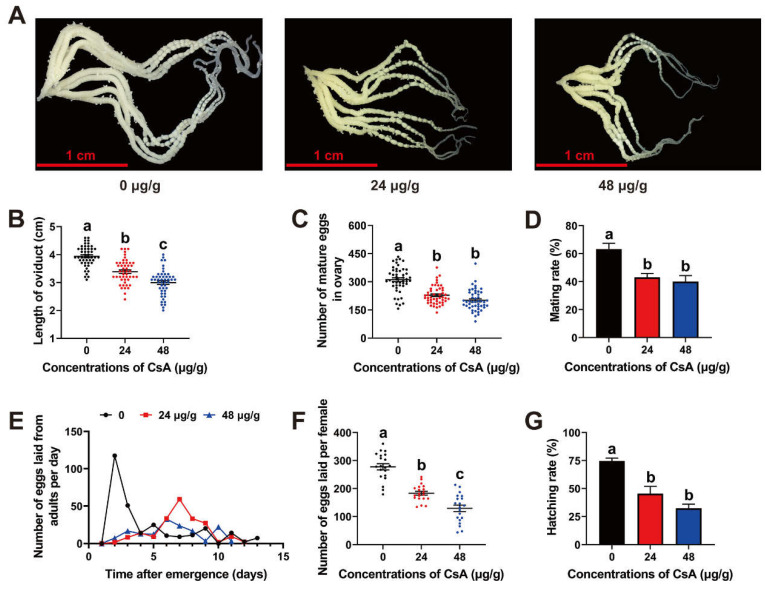
Post-exposure effects of CsA on reproduction of *O. furnacalis*. (**A**) Ovaries under the different treatments of CsA. (**B**) Ovary length under the different treatments of CsA. (**C**) Number of mature eggs in ovaries under each treatment. (**D**) Effects of CsA on mating rate. (**E**) Eggs laid by adults each day under different treatments of CsA. (**F**) Eggs laid per female under different treatments of CsA. (**G**) Hatching rate of eggs under different treatments of CsA. Data are means ± SE of more than three biological replicates. Significant differences were analyzed using ANOVA followed by Tukey’s (equal variances assumed) or Dunnett’s T3 (equal variances not assumed) HSD test on SPSS Statistics 20. Different lowercase letters indicate significant differences at level of *p* < 0.05.

CsA exposure at the larvae stage also affected adult reproduction, significantly suppressing ovarian development (Figure 2A). The length of the oviduct in the control (3.94 cm) significantly decreased to 3.39 (24 μg/g CsA) and 2.99 cm (48 μg/g CsA) (F = 60.55; df = 143; *p* = 0.0001; Figure 2B). The ovaries of the different groups contained 311.31 (control), 228.67 (24 μg/g CsA), and 201.44 (48 μg/g CsA) mature eggs (F = 46.42; df = 143; *p*= 0.0001; Figure 2C). The mating rates of adults in the 24 and 48 μg/g CsA groups were 43.06% and 40.00%, respectively, both of which were significantly lower than that of the control (63.21%; F = 10.96; df = 14; *p* = 0.0020; Figure 2D). In the control, oviposition by adult females peaked 2 days after emergence but was delayed to 7 (24 μg/g CsA) and 6 (48 μg/g CsA) days after the CsA treatment (Figure 2E). The total number of eggs laid by females significantly decreased from 277.65 (control) to 129.25 (48 μg/g CsA) (F = 57.32; df = 59; *p* = 0.0001; Figure 2F). In the F1 generation, only 43.17% (24 μg/g CsA) and 33.03% (48 μg/g CsA) of eggs successfully hatched, which represents markedly lower rates than in the control (67.76%; F = 9.39; df = 32; *p* = 0.0007; Figure 2G).

### 3.5. CsA Inhibits the CaN Activity of O. furnacalis

**Figure 3:**
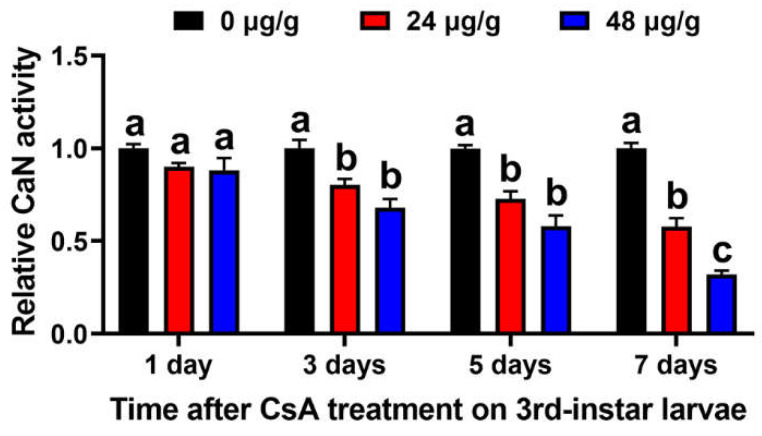
CaN activity of the larval midgut after treatment with CsA for 1, 3, 5, and 7 days. Data are means ± SE of three biological replicates. Different lowercase letters upon error bars indicate significant differences analyzed using ANOVA followed by Tukey’s (equal variances assumed) or Dunnett’s T3 (equal variances not assumed) HSD test on SPSS Statistics 20 at level of *p* < 0.05.

Since CaN is the target of CsA in animals, the CaN activity of the CsA-fed larvae was investigated. The relative CaN activity in the midgut of the larvae treated with 24 and 48 μg/g of CsA for 1 day was lower than that of the control, although the difference was not significant. However, after 3, 5, and 7 days of treatment, CsA significantly inhibited CaN activity in larval midgut (Figure 3). The respective relative CaN activity of the larvae fed 24 and 48 μg/g of CsA was 80.36% and 68.07% of that of the control after CsA treatment for 3 days (F = 23.06; df = 8; *p* = 0.0064); 72.81% and 58.01% after CsA treatment for 5 days (F = 24.70; df = 8; *p* = 0.0013); and 57.71% and 31.91% after CsA treatment for 7 days (F = 68.95; df = 8; *p* = 0.0008).

### 3.6. Combined Toxicity of CsA and Five Insecticides against Third-Instar Larvae

**Figure 4:**
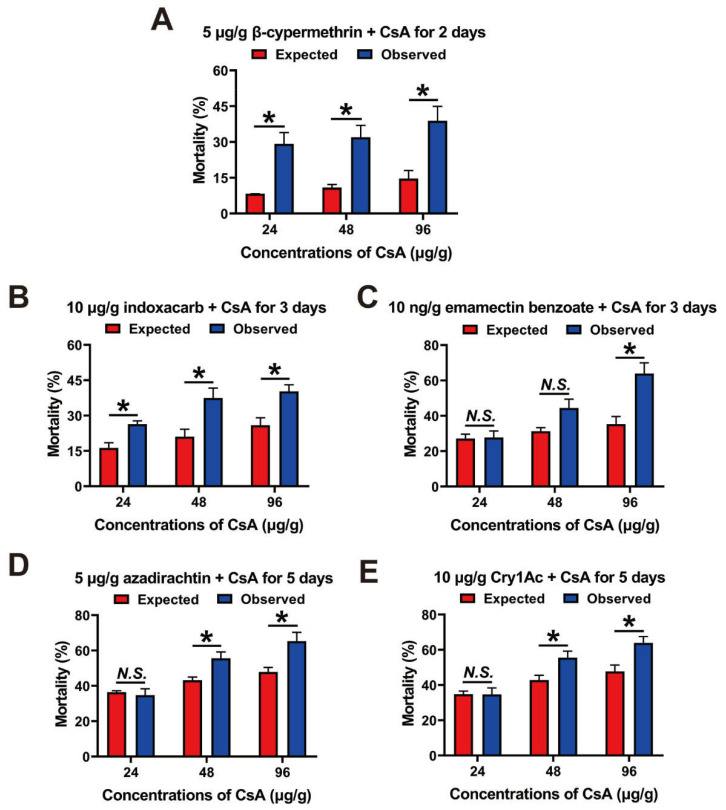
Combined toxicity of CsA and five toxins against 3rd-instar larvae of *O. furnacalis*. (**A**) Significant synergistic effects of the combinations of 5 μg/g of β-cypermethrin + 24/48/96 μg/g of CsA against 3rd-instar larvae for 2 days. (**B**) Significant synergistic combined toxicities of 10 μg/g of indoxacarb + 24/48/96 μg/g of CsA against 3rd-instar larvae for 3 days. (**C**) Expected and observed mortality under the combinations of 10 ng/g of emamectin benzoate + 24/48/96 μg/g of CsA for 3 days. (**D**) Expected and observed mortality caused by the combinations of 5 μg/g of azadirachtin + 24/48/96 μg/g of CsA against 3rd-instar larvae for 5 days. (**E**) Expected and observed mortality of larvae fed 10 μg/g of Cry1Ac + 24/48/96 μg/g of CsA for 5 days. Data are shown as means ± SE of three biological replicates, and the significant differences were analyzed using independent samples *t*-test on SPSS Statistics 20; “*” indicates *p* < 0.05, “*N.S.*” indicates no significant difference.

Four insecticides (5 μg/g of β-cypermethrin, 10 μg/g of indoxacarb, 10 ng/g of emamectin benzoate, and 5 μg/g of azadirachtin) and one Bt toxin (10 μg/g of Cry1Ac) were mixed with CsA (24, 48, and 96 μg/g) to investigate their combined toxicity in 3rd- instar larvae. After applying β-cypermethrin + CsA for 2 days, the observed mortalities of the three combinations were significantly higher than the expected mortality (t = 4.34, df = 4, *p* = 0.012; t = 4.08, df = 4, *p* = 0.015; t = 3.49, df = 4, *p* = 0.025) (Figure 4A). The combined toxicities of the indoxacarb + CsA and the emamectin benzoate + CsA were analyzed after treatment for 3 days. The toxic effects were mainly synergistic. In particular, the expected mortality for 10 μg/g of indoxacarb + 24/48/96 μg/g of CsA was lower than the observed mortalities (t = 3.89, df = 4, *p* = 0.018; t = 3.13, df = 4, *p* = 0.035; t = 3.39, df = 4, *p* = 0.027) (Figure 4B). Similar results were found for the combination of 10 ng/g of emamectin benzoate + 96 μg/g of CsA (t = 3.85, df = 4, *p* = 0.018). However, the expected and observed mortalities were similar for 10 ng/g of emamectin benzoate + 24/48 μg/g of CsA (Figure 4C). The combined toxicities of azadirachtin + CsA and Cry1Ac + CsA were calculated after feeding the toxic diet for 5 days. For the combination of 5 μg/g azadirachtin + 24 μg/g CsA, there was no significant difference between the expected and observed mortalities. However, the observed mortality for the combination of 5 μg/g of azadirachtin + 48/96 μg/g of CsA significantly exceeded the expected mortality (t = 3.01, df = 4, *p* = 0.040; t = 3.09, df = 4, *p* = 0.037) (Figure 4D). Finally, the toxic effects of 10 μg/g of Cry1Ac + 24 μg/g of CsA and 10 μg/g of Cry1Ac + 48/96 μg/g of CsA on third-instar larvae were synergistic: the observed mortalities were significantly higher than the expected mortality (t = 2.78, df = 4, *p* = 0.049; t = 3.11, df = 4, *p* = 0.036) (Figure 4E).

## 4. Discussion

Because CsA is a product of fungal metabolism, in this study, its insecticide activity was compared with that of Bt toxins, which are the most widely used biogenic insecticides in the world for controlling lepidopteran pests, including *O. furnacalis*. Bioassays of laboratory-raised neonatal larvae of *O. furnacalis* revealed that the respective LC_50_ values of Cry1Aa, Cry1Ab, Cry1Ac, and Cry1F were 0.18 (0.15–0.22), 0.28 (0.2–0.36), 0.26 (0.19–0.34), and 0.52 (0.36–0.70) μg/g after 7 days of exposure [34]. Regarding Vip toxins, Vip3Ca is the most toxic protein (LC_50_ = 1.2 µg/g), followed by Vip3_ch2 (LC_50_ = 2.3 µg/g) and Vip3_ch4 (LC_50_ = 3.9 µg/g). The insecticidal activity of Vip3Aa is relatively weak with an LC_50_ of more than 100 µg/g compared with that of toxins mentioned above [35]. However, *O. furnacalis* has developed resistance to various Cry1 toxins, including Cry1Ab, Cry1Ac, Cry1Ah, Cry1F, and Cry1Ie [36,37,38,39]. As an alternative biological insecticide, although CsA has lower toxicity (LC_50_ = 113.02 µg/g, Table 1) than most of these Bt toxins for the newly hatched *O. furnacalis* larvae, in this study, its insecticidal activity was significant. The sublethal effects of CsA on *O. furnacalis* were also obvious, including decreases in the size and weight of larvae and pupae, mating rate, ovary size, and egg production and hatchability (Figure 1 and Figure 2). These insecticides are always considered in integrated pest management strategies due to their potential to suppress the offspring population [40,41]. Therefore, it is important to explore cyclosporin-like insecticides.

It is worth noting that CsA is mass-produced from different species of fungi, including *A. terreus*, *C. columbinum*, *P. fellutanum*, *C. lucidum*, and many *Trichoderma* species, by deep fermentation [15]. These fungi produce CsA derivatives, such as isolated natural cyclosporins (B-I, K-Z, and Cy26-Cy32) and FR901459, which have similar backbone structures and inhibitory activities; their insecticidal activities should be explored [40,41]. In addition, the insecticidal activities of CsA and CsA-like analogs could be improved. Given that the synthesis and modification of CsA can produce many compounds, specific insecticidal activities could be achieved [42,43].

Furthermore, CsA could be used in combination with insecticides to improve the overall toxicity and manage insect resistance. β-cypermethrin, indoxacarb, emamectin benzoate, azadirachtin, and Cry1Ac are widely used to control *O. furnacalis*. β-cypermethrin prevents closure of the sodium-potassium gate and affects the ability of the γ-aminobutyric acid receptor (GABA) to trigger multiple nerve impulses, thereby resulting in excitability and convulsions in target insects [44]. Indoxacarb blocks nerve conduction by inhibiting the entry of Na^+^ into nerve cells [45,46]. Emamectin benzoate acts on GABA and the glutamate-gated chloride channel (Glu-Cl) to increase the amount of Cl^−^ in nerve cells, thereby paralyzing target insects [47,48]. Unlike these three insecticides, azadirachtin and Cry1Ac are digestive system insecticides [49,50,51,52]. Azadirachtin inhibits the activity of digestive enzymes and is an antifeedant [51]. Cry1Ac is a widely used Bt toxin that binds to specific brush border receptors and causes the formation of pores on midgut epithelial cells, thereby killing the insects [52]. The toxic effects in this study were all independent or synergistic (Figure 4), indicating that CsA has a novel insecticidal target and may be used as a synergistic agent of current insecticides. Our experiments also confirmed that CsA inhibited CaN activity in *O. furnacalis* (Figure 3). Importantly, given its unique mechanisms, CsA also has the potential for managing insect resistance. CsA also is an ABC transporter inhibitor [22,23,53,54]. Insect ABC transporters are involved in insecticide detoxification and the mechanism of action of the Bt toxin. Changes in ABC transporters can cause broad insecticide [53,55]. Importantly, CsA is an ABC transporter inhibitor that reversed the resistance to ivermectin toxicity in the cattle tick, *R.* (*Boophilus*) *microplus* [22,23]. These findings indicate that CsA has potential as a synergistic agent of current insecticides and even as a new insecticide for controlling insect resistance.

CsA enters cells and binds to cyclophilin to suppress CaN activity and prevent dephosphorylation of the nuclear factor of activated T-cells [18,56,57]. Cyclophilin and CaN share high amino acid identities in different species (Figures S1 and S2), which indicates the conservation of the CaN function and the CaN pathway. In humans, CsA specifically inhibits CaN, an enzyme activated by Ca^2+^ that plays a key role in immunity [18]. We investigated the role of CsA in *O. furnacalis* by measuring CsA activity. As expected, CaN activity was significantly suppressed by CsA treatment (Figure 3). CaN has not been reported as a target for pest control, although it plays a role in humoral immunity by binding with relish to regulate the expression of *attacin*, *cecropin D*, and *gloverin* in *Helicoverpa. armigera* larvae [58]. While CsA is not toxic to *G. mellonella*, it significantly suppressed the humoral immune response in *G. mellonella*, increasing the mortality due to *Pseudomonas aeruginosa* infection [21,59]. As a result, CsA may suppress humoral immunity by inhibiting CaN activity, thus causing mortality in *O. furnacalis*. The suppression of CaN activity may have other negative effects, such as reducing mating and the insect population (Figure 2), because CaN also functions in pheromone biosynthesis and the mating behavior of lepidopterans [29,31,60]. As a result, CsA is toxic because it targets CaN, making it a good target for pest management. These results indicate that targeting CaN has potential in the design of insecticides for lepidopterans and for use in transgenic RNAi crops to open new avenues for insecticide study.

## 5. Conclusions

The global food shortage and the inevitable development of insecticide resistance necessitate the discovery of new insecticides or insecticidal targets for sustainable management of insect pests. This study provides the first information on the potential of CsA as an insecticide and CaN as an insecticide target to control lepidopterans. However, because the experimental conditions and materials were limited, only pure CsA was used to investigate its insecticidal activity against *O. furnacalis* in the laboratory. CsA is mass-produced by deep fermentation using different species of fungi [14]. Although we suggest that CsA can be used as a microbial insecticide similar to sprayed Bt insecticides, not all the fungal metabolites, CsA analogs, and CsA derivatives were tested because of limited resources. Further studies should explore the toxicity of active CsA-like substances and the safer and more economical ways to apply them.

## Figures and Tables

**Table 1:** Insecticidal activity of CsA against *O. furnacalis* larvae 7 days after feeding.

Larvae	LC_50_ (95% CL^a^) (μg/g)	LC_95_ (95% CL) (μg/g)	Slope ± SE	χ^2^	*p* ^b^
Neonates	113.02 (60.38–289.71)	1,107.64 (381.54–38,337.57)	1.66 ± 0.15	20.44	<0.01
Third-instar	198.70 (134.36–317.63)	2457.28 (1096.90–12,480.19)	1.51 ± 0.14	7.38	0.117

^a^ CL: confidence limit. ^b^ Heterogeneity was used in the calculation of confidence limits when *p* < 0.15.

## Data Availability

The data presented in this study are available on request from the corresponding author.

## Supplementary Materials

The following supporting information can be downloaded at: https://www.mdpi.com/article/10.3390/insects13100965/s1, Figure S1: amino acid sequence alignment for CaNs of *O. furnacalis* and other species; Figure S2: amino acid sequence alignment for cyclophilins of *O. furnacalis* and other species.
