# Supplementary material for: Cyclosporin A as a Potential Insecticide to Control the Asian Corn Borer Ostrinia furnacalis Guenée (Lepidoptera: Pyralidae)
Source: Insects. 2022 Oct 21;13(10):965. doi: 10.3390/insects13100965 (PMC9604310; doi:10.3390/insects13100965)
Supplement: Supplementary file 1 [file insects-13-00965-s001.zip › insects-1878383-supplementary.pdf]

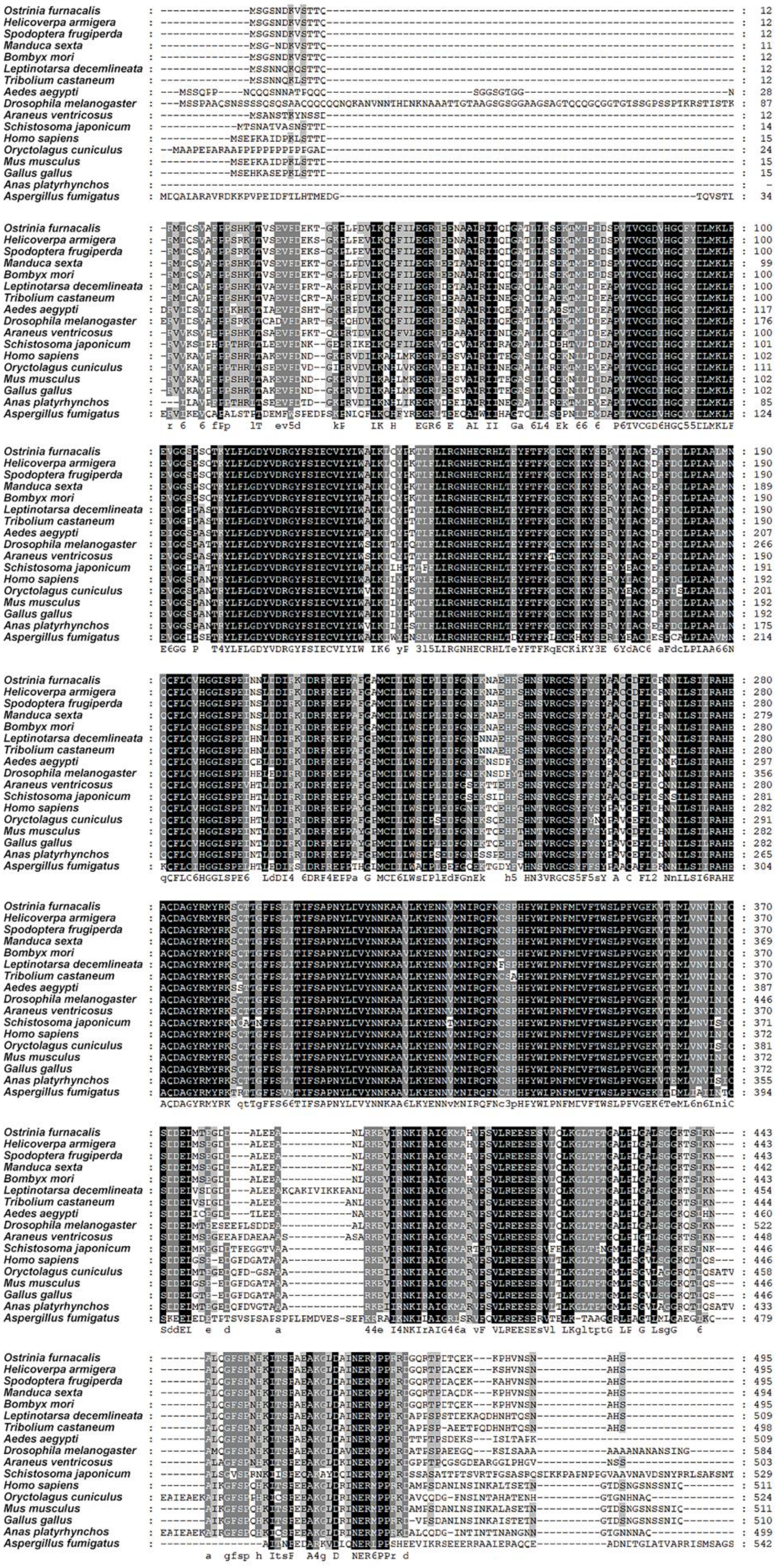
Ostrinia furnacalis
Helicoverpa armigera
Spodoptera frugiperda
Manduca sexta
Bombyx mori
Leptinotarsa decemlineata
Tribolium castaneum
Aedes aegypti
Drosophila melanogaster
Araneus ventricosus
Schistosoma japonicum
Homo sapiens
Oryctolagus cuniculus
Mus musculus
Gallus gallus
Anas platyrhynchos
Aspergillus fumigatus

Figure S1. Amino acid sequence alignment for CaNs of *O. furnacalis* and other species. The black, dark grey, light gray and colourless areas respectively indicate the 90–100%, 80–90%,60–80% and lower than 60% similarity/identity. Specific names and GenBank IDs: *O. furnacalis*, XP_028156950.1; *Helicoverpa armigera*, XP_021181213.1; *Spodoptera frugiperda*, XP_035436316.1; *Manduca sexta*, XP_030032629.1; *Bombyx mori*, NP_001037025.1; *Leptinotarsa decemlineata*, XP_023017364.1; *Tribolium castaneum*, EEZ99522.2; *Aedes aegypti*, *XP_021707011.1*; *Drosophila melanogaster*, NP_001259622.1; *Araneus ventricosus*, GBL91594.1; *Schistosoma japonicum*, KAH8863363.1; *Homo sapiens*, NP_001124163.1; *Oryctolagus cuniculus*, NP_001076196.1; *Mus musculus*, NP_001280551.1; *Gallus gallus*, XP_040527134.1; *Anas platyrhynchos*, EOB04152.1; *Aspergillus fumigatus*, KAF4282405.1.

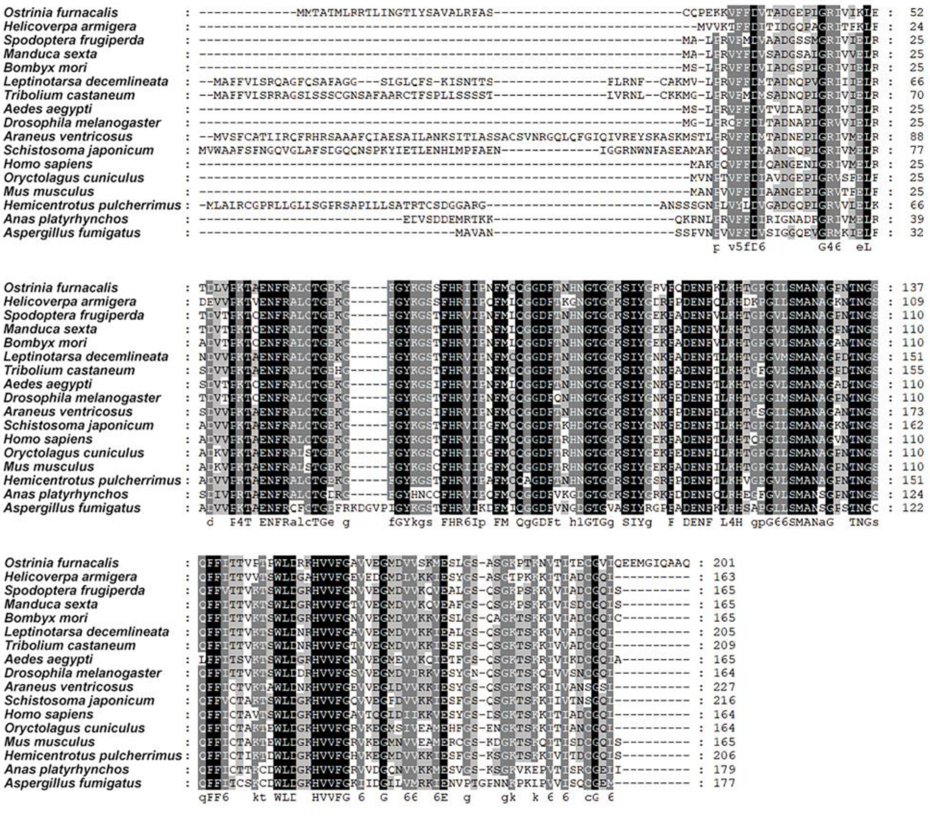

Figure S2. Amino acid sequence alignment for cyclophilins of *O. furnacalis* and other species. The similarity/identity of amino acid sequences of 90–100%, 80–90%, 60–80% and lower than 60% were respectilely displayed with black, dark grey, light gray

and colourless areas. Specific names and GenBank IDs: *O. furnacalis*, XP_028172843.1; *H. armigera*, XP_021200657.1; *S. frugiperda*, XP_035453679.1; *M. sexta*, XP_030031426.1; *B. mori*, NP_001037301.1; *L. decemlineata*, XP_023012814.1; *T. castaneum*, EEZ98965.2; *A. aegypti*, ABF18058.1; *D. melanogaster*, NP_523366.2; *Araneus ventricosus*, GBL90912.1; *S. japonicum*, AAA29863.1; *H. sapiens*, AAC51927.1; *O. cuniculus*, AAF22215.1; *M. Musculus*, NP_598845.1; *Hemicentrotus pulcherrimus*, AAB37708.1; *A. platyrhynchos*, AJR16770.1; *A. fumigatus*, CAI78448.1.
